# Conotoxin kM-RIIIJ reveals interplay between K_v_1-channels and persistent sodium currents in proprioceptive DRG neurons

**DOI:** 10.1038/s41598-024-82165-5

**Published:** 2024-12-28

**Authors:** Shrinivasan Raghuraman, Jackson Carter, Markel Walter, Manju Karthikeyan, Kevin Chase, Matías L. Giglio, Mario Giacobassi, Russell W. Teichert, Heinrich Terlau, Baldomero M. Olivera

**Affiliations:** 1 School of Biological Sciences, University of Utah, Salt Lake City, Utah USA; 2 Institute of Physiology, Christian-Albrechts-University Kiel, 24118 Kiel, Germany

**Keywords:** Neuroscience, Molecular neuroscience

## Abstract

Voltage-gated potassium channels (VGKCs) comprise the largest and most complex families of ion channels. Approximately 70 genes encode VGKC alpha subunits, which assemble into functional tetrameric channel complexes. These subunits can also combine to form heteromeric channels, significantly expanding the potential diversity of VGKCs. The functional expression and physiological role of heteromeric K-channels have remained largely unexplored due to the lack of tools to probe their functions. Conotoxins, from predatory cone snails, have high affinity and specificity for heteromeric combinations of K-channels and show great promise for defining their physiological roles. In this work, using conotoxin kM-RIIIJ as a pharmacological probe, we explore the expression and physiological functions of heteromeric K_v_1.2 channels using constellation pharmacology platform. We report that heteromers of K_v_1.2/1.1 are highly expressed in proprioceptive neurons found in the dorsal root ganglion (DRG). Inhibition of K_v_1.2/1.1 heteromers leads to an influx of calcium ions, suggesting that these channels regulate neuronal excitability. We also present evidence that K_v_1.2/1.1 heteromers counteract persistent sodium currents, and that inhibiting these channels leads to tonic firing of action potentials. Additionally, kM-RIIIJ impaired proprioception in mice, uncovering a previously unrecognized physiological function of heteromeric K_v_1.2/1.1 channels in proprioceptive sensory neurons of the DRG.

## Introduction

The K-channel superfamily is the largest and most diverse class of ion channels. Because of their central role in determining the dynamics of voltage changes across cell membranes, a characterization of voltage-gated K-channels (VGKC) is key to understanding cellular function in different systems including the nervous, endocrine, and immune systems. Approximately 70 distinct mammalian genes encode K-channel subunits^1^. The functional ion-channel complex requires 4 principal α-subunits to assemble a pore, resulting in a vast number of potential combinatorial possibilities. It has been shown that heteromeric combinations of different subunits often result in emergent properties which cannot be predicted from the phenotypes of the corresponding homomeric K-channel complexes^2,3^. Furthermore, the properties of these channels are highly cell-type specific and depend on the constellations of ion channels and receptors that are co-expressed in the same cell. In sensory neurons of the dorsal root ganglia (DRG), channels of K_v_1 family act as excitability brakes and regulate mechanosensation, thermosensation, and nociception^4–6^. Although powerful molecular genetic methods have been developed in recent years, the functional expression and roles of heteromeric K-channels have remained underexplored. Genetic knockout strategies are ineffective for studying heteromeric ion channels because knocking out one gene eliminates all homomeric and heteromeric combinations that contain the encoded K channel subunit, complicating the analysis of heteromer function. Thus, the study of heteromeric ion channels require unique pharmacological tools that can dissect and discriminate between K-channel isoforms.

Cone snail venoms are one of the rich sources of pharmacological tools and have shown promising potential to discriminate between closely related subtypes of ion channels and receptors^7^. Due to their high selectivity profile, conopeptides are sought-after pharmacological agents in ion channel research and have direct diagnostic and therapeutic potential. In this work, we use one of the conopeptides, kM-RIIIJ, which has been well characterized chemically and biochemically^8^. We previously established that conotoxin kM-RIIIJ, isolated from *Conus radiatus*, selectively discriminates between heteromeric combinations of K_v_1.2 channels^9^. The toxin has high affinity for K-channel composed of three subunits of K_v_1.2 and one subunit of either K_v_1.1 or K_v_1.6, with an IC_50_ in the low nanomolar range observed in a heterologous expression system^9^. This provides a unique opportunity to study the functional expression and biological roles of these heteromeric K-channels in native neuronal cells.

To assess the expression and function of heteromeric K_v_1.1/1.2 channels, we used a calcium imaging based platform that employs highly selective pharmacological agents (“Constellation Pharmacology”) in combination with molecular genetics, single-cell transcriptomics, and whole-cell electrophysiology. Constellation pharmacology tracks the constellation of ion channels and receptors expressed in individual neuronal cell types, facilitating the study of membrane macromolecules that work in concert to shape cellular physiology^10^. Using this approach, we monitored the bioactivity of conopeptide kM-RIIIJ on various sensory cell types of the mouse DRG. We observed that inhibiting the K_v_1.1/1.2-channel heteromers with the conotoxin kM-RIIIJ elevated intracellular calcium levels in a specific subpopulation of DRG neurons- the proprioceptive neurons^11^. Here we characterized the molecular mechanisms underlying this phenotype and uncovered a novel interplay between voltage-gated sodium and potassium channels in maintaining the resting membrane potential in proprioceptive DRG neurons. Voltage-gated sodium channels can give rise to different types of inward currents including fast transient Na^+^ current (I_NaT_) and non-inactivating persistent Na^+^ current (I_NaP_)^12^. While I_NaT_ underlies the rising phase of action potential, I_NaP_ activates at subthreshold voltages and influences membrane excitability^12,13^. Our work reveals that disrupting the intricate balance between persistent sodium currents and heteromeric K_v_1.1/1.2 channels by conotoxin kM-RIIIJ leads to hyperexcitability in proprioceptive DRG neurons and impaired proprioception, uncovering a novel function of heteromeric K-channels and persistent sodium currents in proprioceptive sensory physiology.

## Results

### kM-RIIIJ elevates intracellular calcium levels in proprioceptive DRG neurons

**Fig.1:**
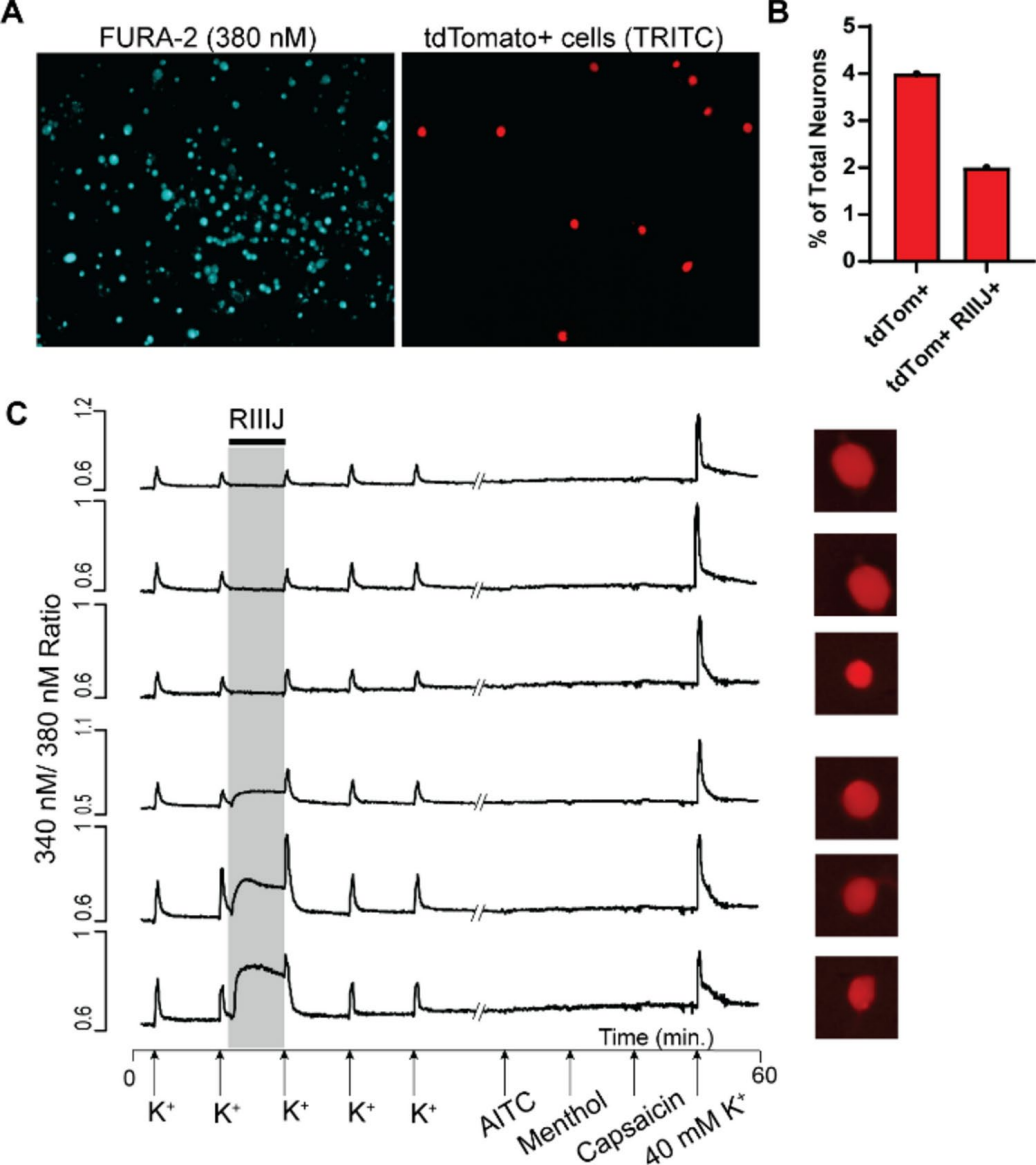
kM-RIIIJ elevates intracellular calcium in a subset of proprioceptive DRG neurons. (**A**). Fluorescent images of DRG cells in primary culture loaded with FURA-2. A subset of these neurons express Td-tomato (labeling proprioceptive DRG neurons). (**B**). Graph showing the percentage of total neurons in culture that express fluorescent td-tomato. 4% of total neurons were labeled, and about 50% of the labeled neurons respond to the application of kM-RIIIJ. (**C**). Representative calcium traces from six proprioceptors. X-axis indicates the application of pharmacological stimulus presented during an hour-long experiment. Y-axis represents ratiometric signal from 340 nm/380 nm excitation wavelengths. kM-RIIIJ elevated intracellular calcium levels in a subset of proprioceptors, as exemplified by bottom three traces.

We previously demonstrated that kM-RIIIJ elevates intracellular calcium levels in proprioceptors and large diameter Aδ-low threshold mechanoreceptors (Aδ-LTMRs)^11^. The effects of kM-RIIIJ were monitored at various concentrations (10 nM, 100 nM, and 1 μM) on a heterogeneous population of sensory DRG neurons obtained from transgenic mice specifically generated to label proprioceptive neurons^14^. These mice were created by crossing Pvalb-Cre mice with Ai14 reporter mice to drive the expression of td-Tomato in proprioceptors^15^. As shown in Fig. 1A, all cells loaded with calcium indicator FURA-2 fluoresce at excitation wavelength of 380 nm. Only a small subset (~ 4% of total neurons) express td-Tomato (visualized when excited at 546 nm light). We observed that 100 nM kM-RIIIJ induced an influx of calcium ions specifically in a subset of proprioceptors. As shown in Fig. 1B, 50% of the td-Tomato^+^ cells show kM-RIIIJ induced calcium elevation. Representative calcium traces from individual neurons are shown in Fig. 1C. The application of 100 nM kM-RIIIJ elevated intracellular calcium in a subset of neurons (bottom three traces) but not other cells (top three traces). This data suggests that the expression of K_v_1 channels comprising K_v_1.2 and K_v_1.1 or K_v_1.6 are crucial for maintaining calcium homeostasis and regulating the function of proprioceptors.

To identify which of the potential subunits are expressed in these DRG neurons, we picked individual kM-RIIIJ-sensitive proprioceptors and performed single-cell transcriptomic analysis in tandem with constellation pharmacology experiments. Supplementary Table 1 shows the levels of transcripts expressed by n = 23 proprioceptors affected by kM-RIIIJ. As shown, we detected high levels of K_v_1.1 and K_v_1.2, in contrast, the levels of K_v_1.6 were either relatively low or absent, reducing the possibility of heteromeric K_v_1.2 channels containing K_v_1.6 subunits. To test the functional expression K-channel subunits, we used K_v_1.6 blocker (conotoxin kJ-PlXIVA) and K_v_1.1 blocker (dendrotoxin-K)^16,17^. As shown in Supplementary Figure S1, proprioceptors sensitive to the application of kM-RIIIJ were not affected by the application of kJ-PlXIVA. Fig.S1B are traces from neurons within the same experiment that displayed disturbed calcium baseline in the presence of 16 μM PlXIVa, suggesting high expression of functional K_v_1.6 subunit containing K-channels in other neurons in culture. Additionally, as shown in supplementary Figure S2, application of 100 nM dendrotoxin-K caused disturbance to calcium levels in proprioceptors. We observed that RIIIJ-sensitive proprioceptors display spectrum of response to the application of dendrotoxin-K, indicating differential expression or density of K_v_1.1 containing channels. Taken together, the results demonstrate that K_v_1 heteromers, likely composed of K_v_1.2 and K_v_1.1 subunits, are expressed in proprioceptive DRG neurons and inhibiting heteromeric K_v_1 channels with kM-RIIIJ induces calcium influx in proprioceptors.

### kM-RIIIJ blocks 4-AP sensitive (and not TEA-sensitive) K-channels and the effects of kM-RIIIJ are abolished in the presence of tetrodotoxin

**Fig.2:**
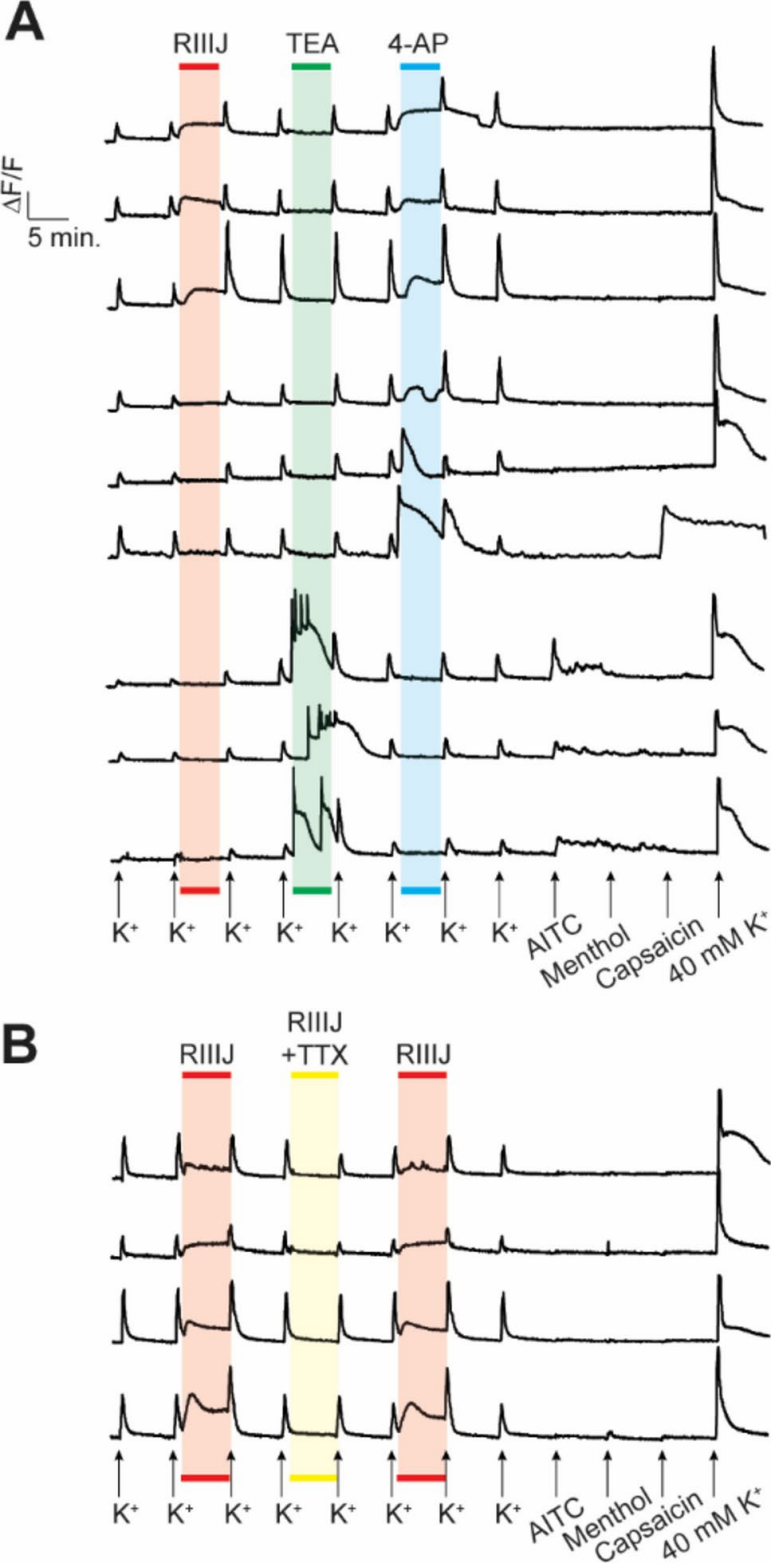
(**A**). kM-RIIIJ blocks a subset of 4-AP sensitive (but not TEA sensitive) K-channels. Representative calcium traces from 3 cells per group. Top three traces are from proprioceptors, which respond to the application of kM-RIIIJ and 4-AP, but not TEA. Middle traces are other non-proprioceptive DRG neurons affected by 4-AP, but not kM-RIIIJ. Bottom three traces are representative traces from cells that respond to the application of TEA, but not kM-RIIIJ or 4-AP (**B**). TTX abolishes the phenotypic effects of kM-RIIIJ. kM-RIIIJ induced elevation of intracellular calcium was abolished by the co-application with 1 μM TTX.

We compared the effects of kM-RIIIJ with two broad-spectrum blockers of K-channels- 4-aminopyridine (4-AP) and tetraethyl ammonium chloride (TEA). 4-AP is known to block transient, fast activating and inactivating K-currents (I_A_-currents), which are essential for controlling the timing of action potential firing and shaping the neuronal response to inputs^18,19^. On the other hand, TEA inhibits delayed rectifiers (I_DR_) which are crucial for resetting the neuronal membrane potential after an action potential and for controlling the overall action potential frequency^20^. As shown in Fig. 2A, the phenotypic effects of kM-RIIIJ on proprioceptors were analogous to those elicited by the application of 1 mM 4-AP, while no effect was elicited by 10 mM TEA. Other non-proprioceptive neurons (bottom three traces) were sensitive to the application of TEA, however, these neurons did not respond to the application of kM-RIIIJ or 4-AP. The middle three traces are from other non-proprioceptive sensory DRG neurons that were sensitive to the application of 4-AP, but not kM-RIIIJ. These observations suggest that kM-RIIIJ blocks a subset of 4-AP sensitive K-channels, but not TEA sensitive K-channels. Furthermore, the presence of 1 μM TTX, a potent blocker of voltage-gated sodium channels, abolished the phenotypic rise in intracellular calcium elicited by kM-RIIIJ, suggesting a mechanistic interplay between the TTX-sensitive sodium channels and kM-RIIIJ-sensitive potassium channels in proprioceptors (Fig. 2B).

### kM-RIIIJ blocks K-currents in proprioceptive DRG neurons, resulting in tonic firing of action potentials

**Fig.3:**
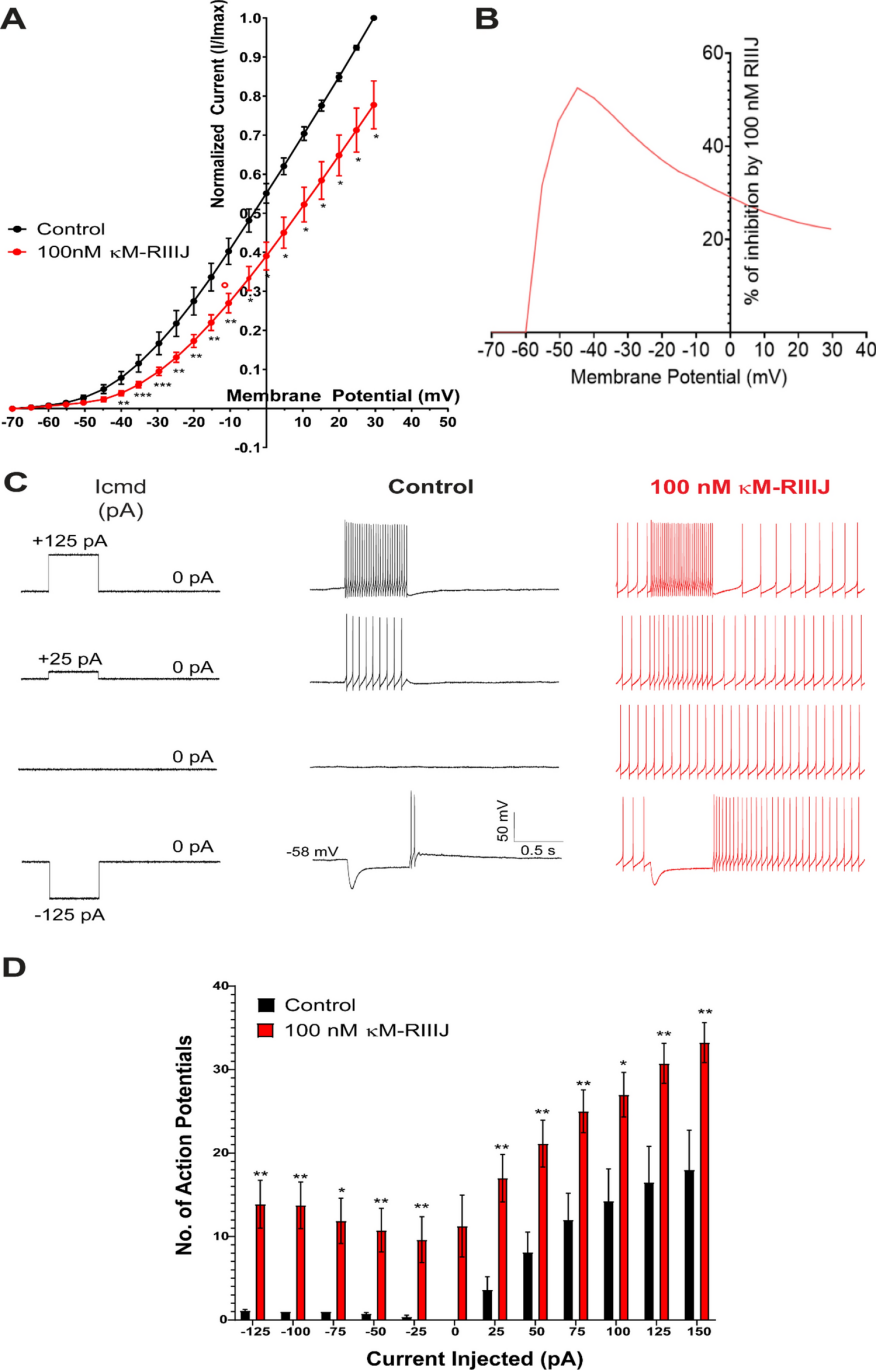
kM-RIIIJ blocks K-currents in proprioceptors and result in tonic firing of action potentials. (**A**). Whole-cell K-currents recorded from kM-RIIIJ-sensitive proprioceptors. As shown, 100 nM kM-RIIIJ significantly inhibited whole-cell K-currents. (**B**) shows the percentage of whole cell K-currents inhibited by 100 nM kM-RIIIJ across membrane potentials ranging from -70 mV to + 30 mV. (**C**). Whole-cell current clamp experiments were performed on kM-RIIIJ-sensitive proprioceptors. With depolarizing or hyperpolarizing current injection, cells exhibit tonic firing after the current injection is terminated. Note the tonic activity in the presence of kM-RIIIJ in the absence of any current injections (third panel). (**D**) The number of action potentials recorded in response to current injections ranging from -125pA to + 150pA is shown. n = 10 proprioceptors sensitive to kM-RIIIJ. Whole-cell voltage clamp or whole-cell current clamp experiments were performed in-tandem with calcium imaging experiments to identify kM-RIIIJ-sensitive proprioceptors. * *p* < 0.05, ** *p* < 0.01, *** *p* < 0.001 determined by multiple paired *t*-test.

We next focused on characterizing whole-cell K-currents blocked by kM-RIIIJ. We performed whole-cell voltage clamp experiments on proprioceptors DRG neurons that were affected by 100 nM kM-RIIIJ. The whole-cell K-currents were monitored by subjecting the cells to a ramp protocol, elevating the membrane potential from −70 mV to + 30 mV at the rate of 2.5 mV/s. The ramp protocol was selected due to its ability to mimic the gradual changes in membrane potential that occur physiologically, thus providing insights into the kinetic properties of channel activation and inactivation. Additionally, this protocol allows for the concurrent observation of persistent sodium currents, offering a comprehensive view of the ion dynamics within these neurons. The ramp protocol also provides information on currents over whole-voltage range in one test pulse, in addition to inactivating fast sodium currents^21^. Whole-cell K-currents were recorded in the presence and absence of 100 nM kM-RIIIJ. As shown in Fig. 3A, 100 nM kM-RIIIJ blocked K-currents across the range of membrane potentials tested. Figure 3B shows the percentage of whole-cell K-currents blocked at different membrane potentials. As shown, 100 nM kM-RIIIJ blocked about 50% of K-currents at −40 mV and ~ 20% of total K-currents at positive membrane potential of + 30 mV (n = 10 kM-RIIIJ sensitive proprioceptors, p-val < 0.05 using paired t-test).

To test the resulting effects of blocking K-currents on proprioceptors, whole-cell current clamp experiments were performed. As shown in Fig. 3C, changes to membrane potential were recorded in response to the pulse protocol (Icmd). On average, proprioceptors had a resting membrane potential of -56 mV. The application of 100 nM kM-RIIIJ elicited tonic firing of action potentials and the frequency of firing increased with positive current injections. (n = 10 proprioceptors). The observed blockade of K-currents by kM-RIIIJ correlates with a marked increase in neuronal excitability, as evidenced by the induction of tonic action potential firing (Fig. 3D). Thus, heteromeric K_v_1.1/1.2 channels play a regulatory role in maintaining the excitability threshold of proprioceptive neurons. The data also indicates the presence of a persistent depolarizing drive, which is normally counteracted by K_v_1.1/1.2 channels, and causes tonic firing of action potentials when K_v_1.1/1.2 channels are blocked.

### Persistent sodium currents observed in proprioceptive DRG neurons

**Fig.4:**
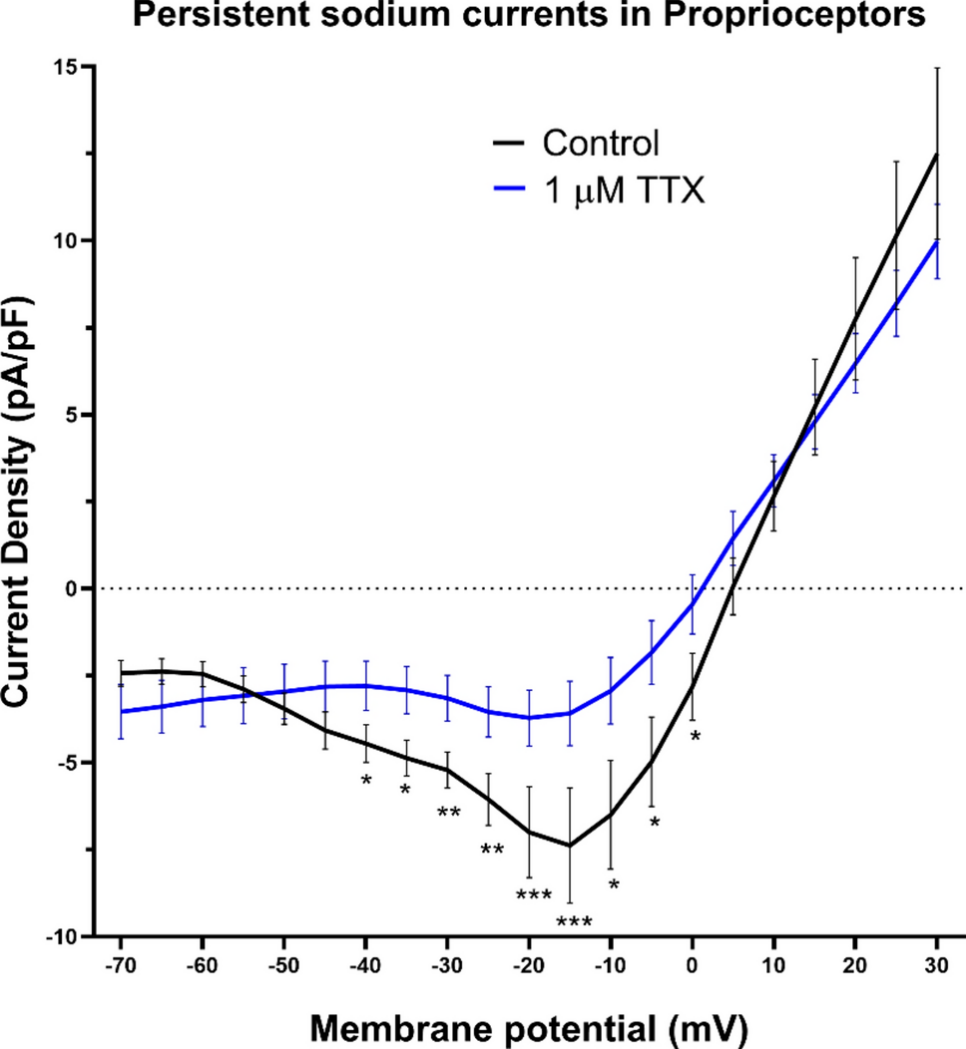
Persistent sodium currents in proprioceptors. Cells were subjected to a ramp protocol elevating the membrane potential from -70 mV to + 30 mV at the rate of 2.5 mV/s. As shown, persistent sodium currents (peaked at -20 mV) were observed, which were inhibited by the application of 1 μM tetrodotoxin (blue trace). Data obtained from n = 11 proprioceptive DRG neurons. External solution contains 10 mM TEA and 1 mM 4-AP to obtain maximum density of I_NaP_. * *p* < 0.05, ** *p* < 0.01, *** *p* < 0.001 determined by using multiple unpaired *t*-test.

Our observations pointed to the presence of persistent inward drive at subthreshold potentials that initiated the tonic firing of action potentials when K_v_1.1/1.2 channels are blocked. As TTX abolished kM-RIIIJ induced calcium influx (Fig. 2B), we investigated whether proprioceptors expressed persistent component of sodium currents (I_NaP_). kM-RIIIJ sensitive proprioceptors were subjected to a ramp protocol elevating the membrane potential from −70 mV to + 30 mV at the rate of 2.5 mV/s and whole-cell sodium currents were recorded. These experiments were performed in the external solution containing 10 mM TEA and 1 mM 4-AP to block all K-currents and to maximize the density of persistent sodium currents. As shown in Fig. 4, we detected persistent sodium currents in proprioceptive DRG neurons (n = 11 proprioceptive DRG neurons), which peaked at −15 mV, with a current density of −7.4 ± 5.4 (pA/pF ± S.D.) and these currents were blocked by the application of 1 μM TTX. These experiments confirmed the presence of persistent sodium currents in proprioceptive DRG neurons, which could potentially drive the cells to tonic firing states in the presence of kM-RIIIJ.

### K_v_1.1/1.2 counteracts persistent sodium currents as revealed by kM-RIIIJ

**Fig.5:**
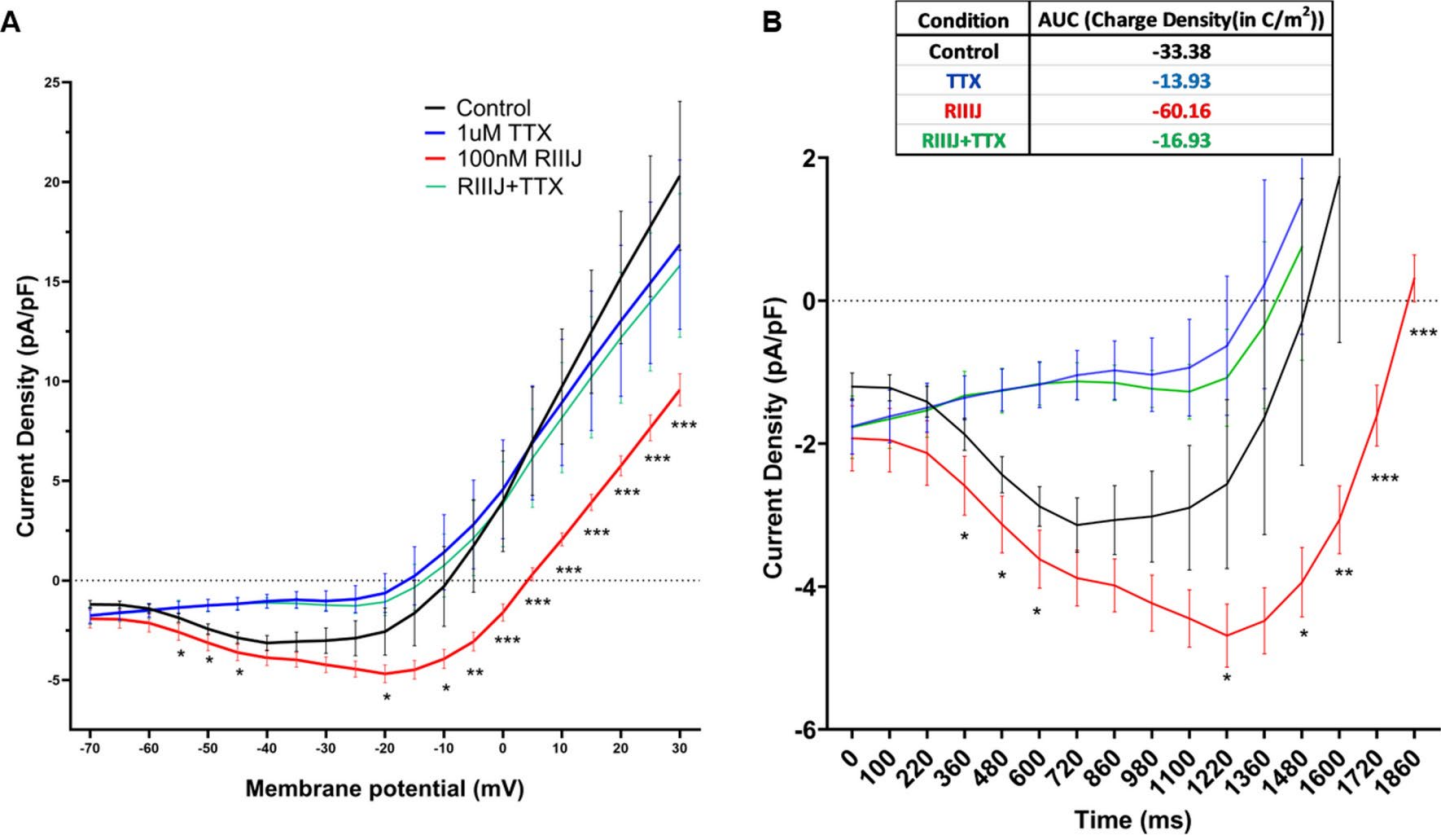
(**A**). kM-RIIIJ increases the influx of persistent sodium currents for the duration of ramp. Application of 100 nM kM-RIIIJ (red trace) significantly increased influx of persistent sodium currents for the duration of the ramp protocol by 14% in the range of -20 mV to + 5 mV compared to control (black trace). The effects were abolished in the presence of 1 μM TTX (blue and green trace). (**B**). 100 nM kM-RIIIJ increased the influx of charge density from -33.38 C/m^2^ (control black trace) to -60.16 C/m^2^ (red trace). n = 15 proprioceptor DRG neurons. *p < 0.05, ** *p* < 0.01, *** *p* < 0.001 determined by Wilcoxon matched-pairs signed rank test.

To monitor the interplay between TTX-sensitive persistent sodium currents and kM-RIIIJ-sensitive K-currents, we conducted ramp experiments using similar external solution as before, but omitted 4-AP (as kM-RIIIJ blocked the 4-AP sensitive K-currents). As shown in Fig. 5A, under control conditions, the persistent sodium currents peaked at −40 mV with a current density of −3.1 ± 1.5 (pA/pF ± S.D., n = 15 proprioceptor neurons). In the presence of 100 nM kM-RIIIJ, the persistent sodium currents peaked at -20 mV with a density of −4.7 ± 1.6 (pA/pF ± S.D.). These data suggest that kM-RIIIJ did not have significant effects on the peak amplitude of persistent sodium currents. However, the application of 100 nM kM-RIIIJ significantly prolonged the influx of persistent sodium currents by 14% for the duration of the ramp protocol, particularly between the range of −20 mV and + 5 mV. This increase in the influx of persistent sodium currents was completely abolished when 1 μM TTX) was co-applied with 100 nM kM-RIIIJ (green trace).

To further understand the physiological impact of this interplay, we plotted the data as current density v/s time plot and calculated the area under the curve to obtain charge density. As shown in Fig. 5B, 100 nM kM-RIIIJ increased the inward positive charge density from -33.38 C/m^2^ to -60.16 C/m^2^. This demonstrates that the block of K_v_1.1/1.2 heteromeric channels increased the inflow of persistent positive charges at membrane potentials where K-channels would normally open and counteract this inflow. This enhanced influx of persistent sodium currents drives depolarization and tonic action potential firing in proprioceptors. Thus, the RIIIJ-sensitive K_v_1.1/1.2 channels seem to provide a counterbalancing hyperpolarizing influence, stabilizing the neuron’s resting potential and preventing runaway excitability by TTX-sensitive persistent sodium currents.

### Injection of kM-RIIIJ impairs proprioception in mice and zebrafish

**Fig. 6:**
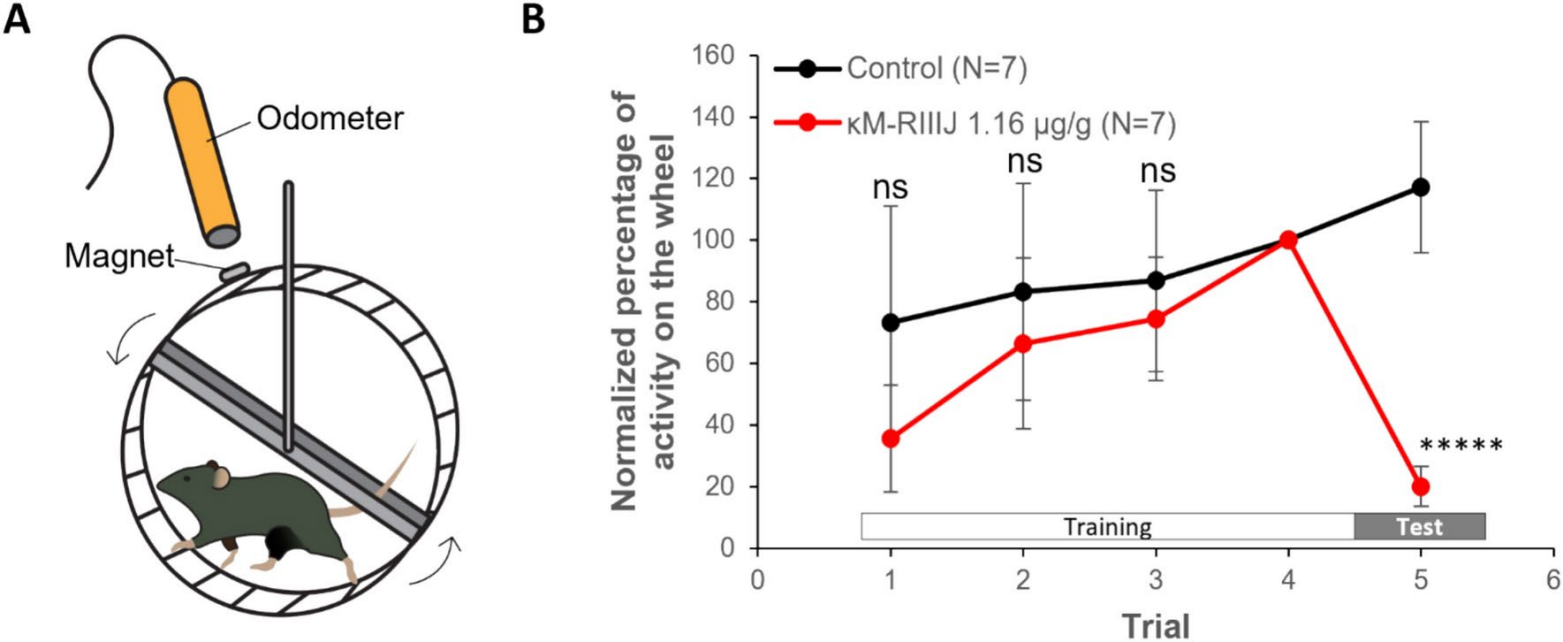
Effect of kM-RIIIJ on locomotion of mice. (**A**). Assay setup. Animals were given a daily i.p. injection of saline solution. 30 min post injection, animals were placed in a chamber with a running wheel coupled to an electromagnetic odometer. Animals were trained on running wheel for 4 days. On day 5, animals were injected with either saline or kM-RIIIJ 30 min prior final trial. (**B**). Normalized percentage of activity. The number of turns on day 4 was considered as 100%. The toxin injected group was compared to the controls using a multiple unpaired t test. ***** *p* < 0.00001. ns = not significant.

To assess the behavioral effects of blocking K_v_1.1/1.2, we performed intraperitoneal injection of kM-RIIIJ in mice. Given the association between impaired coordination and performance in voluntary running, we monitored locomotion and balance using voluntary running wheel assays in mice^22^. Mouse locomotion was monitored both on and off the running wheel. As shown in Fig. 6, injection of 10 nmol kM-RIIIJ (1.16 ± 0.05 mg/kg) significantly reduced the running activity by 80% (*P* < 0.00001, *t*-test). During the assay, mice injected with kM-RIIIJ showed mild signs of uncoordinated locomotion. All mice injected with kM-RIIIJ showed sporadic stumbles when running, usually leading to a deceleration or even a pause in the locomotion. In addition, we observed that the tails of the animals injected with kM-RIIIJ would hit the running wheel multiple times while running (a qualitative indication of uncoordinated proprioception). These observations starkly contrasted the control groups, which showed no stumbling, and positioned their tails parallel to the running wheel during the running sessions, almost never touching the wheel. These behavioral phenotypes suggest that kM-RIIIJ is potentially impairing proprioception in mice.

Given that the conopeptide kM-RIIIJ is isolated from fish hunting cone snail (*C. radiatus*), we speculated that kM-RIIIJ impairs swimming behavior in fish to enable efficient prey capture by cone snails. As shown in supplementary Table 2 (and accompanying videos), injection of kM-RIIIJ impaired swimming behavior in fish. Fish injected with kM-RIIIJ displayed belly up swimming behavior and death. These observations also imply that kM-RIIIJ is an evolutionary adaptation in *C. radiatus* to assist in prey capture by impairing proprioception through the targeting of heteromeric K_v_1.1/1.2 channels.

## Discussion

The constellation of ion channels plays a crucial role in shaping cell type-specific physiology by dictating how cells respond to various stimuli and maintain homeostasis. Each cell type expresses a unique repertoire of ion channels, which together influence its electrical excitability, intracellular signaling, and interactions with the external environment. For instance, neurons may have a specific array of voltage-gated sodium and potassium channels that enable rapid action potentials and precise signal transmission, while cardiac myocytes exhibit a distinct set of ion channels essential for coordinating rhythmic heartbeats. This tailored expression of ion channels ensures that each cell type can perform its specialized functions effectively and adapt to the physiological demands placed upon it. However, the study of ion channels working in concert is impeded by the lack of tools to study diverse heteromeric subtypes of ion channels. In particular, the heteromers of K-channels are underexplored and this study uncovers significant roles of heteromeric K_v_1.1/1.2 channels.

Previous studies suggested a role for K_v_1.1/1.2 channels as an excitability brake in DRG neurons to sensory inputs of touch, pain and thermosensation^4,5,23^. These prior studies demonstrated that blocking K_v_1.1/1.2 channels reduced the threshold for firing action potential when the sensory stimulus is presented. In the present study, we discovered that blocking the heteromers of K_v_1.1/1.2 drives the proprioceptors to a hyperexcitable state, exemplified by tonic spiking behavior of these cells (Fig. 3C), rendering the proprioceptive circuitry non-functional. In the absence of any evoked sensory stimulus, the interplay between heteromeric K_v_1.1/1.2 channels and TTX-sensitive sodium channels are crucial in maintaining the resting membrane potential. Modulating this interaction using conotoxin kM-RIIIJ resulted in impaired sensory/motor coordination, and compromised locomotion (swimming in fish or running in mice, Fig. 6). Although the voluntary running wheel assay captures the cumulative physiological effects of modulating multiple cell types, including proprioceptors, the deficit in locomotion resulting in impaired swimming in fish has a decisive consequence with respect to predator–prey interactions of the cone snail and its fish prey.

This study paves the way for exploring how the interaction between potassium and sodium channels can impact other members of the ion channel constellation, such as Piezo2 receptors, which are crucial for detecting muscle stretch and can modify cellular firing patterns upon activation. Piezo2 channels are crucial mechanoreceptors necessary for normal proprioceptive function. As skeletal muscles contract, Piezo2 channels respond, and the change in membrane depolarization presumably results in the graded response shown in Fig. 3C, control panel. However, kM-RIIIJ completely disrupts the normal graded changes in the number and frequency of action potentials, rendering the proprioceptive circuitry non-functional. Our findings offer new opportunities to investigate the co-dependence of Piezo2 receptors on the interaction between TTX-sensitive sodium channels and kM-RIIIJ-sensitive K_v_1 heteromeric channels in maintaining proper proprioceptor physiology.

## Material and methods

### Animals

The studies were approved by the University of Utah institutional animal care and use committee (IACUC) guidelines (protocol#1627). All methods were carried out in accordance with AAALAC guidelines and reported in accordance with ARRIVE guidelines. 30–60-day old mice (mixed gender) were used for all in vitro studies. Male animals were used for voluntary running wheel assays. Pvalb-IRES-Cre were purchased from Jackson laboratories (Strain code: 017320). The males were crossed with Ai14D female mice purchased from Jackson laboratories (Strain code: 007914). The resulting pups were used for in vitro experiments. In some experiments, animals obtained from transgenic mice (Tg(Calca-EGFP)FG104Gsat) crossed with wild type CD1 females were used. Animals were euthanized by CO_2_ asphyxiation and dorsal root ganglion tissues from lumbar region L1-L6 were harvested to prepare primary cell cultures. 60 day old zebrafish were used for intramuscular injection and testing the effects of kM-RIIIJ on swimming behavior.

### Cell culture and sample preparation

Neuronal cells were cultured according to standard protocols. L1-L6 DRGs were digested using 0.25% trypsin in calcium-free Hanks Balanced Salt Solution (HBSS) in 37 °C water bath for 20 min. Trypsin digestion was quenched using minimal essential media (MEM) supplemented with 10% FBS. Glass Pasteur pipettes were fire polished to different diameters and were used to mechanically triturate the tissues. Cells were filtered through 70-micron mesh, centrifuged at 300 χg force for 10 min and plated on plated on poly-D-lysine-coated glass coverslips. A silicon ring with an inner diameter of 3 mm was placed on coverslips to concentrate cells in a small area and cell suspension was placed in the center of the ring. Cells were supplemented with MEM + 10% FBS an hour after plating and grown overnight at 37 °C in a humidified atmosphere of 5% CO_2_. Experiments were performed between 16 to 24 h after plating.

### Constellation pharmacology

Cells were loaded with 2.5 μM FURA-2 for an hour and calcium imaging was performed to uncover constellation of ion channels and receptors expressed by different DRG neurons. We employed calcium imaging based constellation pharmacology to identify cell types and dissect the interactions between heteromeric potassium channels and persistent sodium currents. Pharmacological agents, allyl iso-thiocyanate (AITC), menthol and capsaicin were purchased from Sigma. kM-RIIIJ was synthesized in our laboratory as described before. Cells were depolarized using 20–25 mM KCl. External buffer (DRG observation solution), contained (in mM): 145 NaCl, 5 KCl, 2 CaCl_2_, 1 Na-citrate, 1 MgCl_2_, 10 HEPES, 10 glucose, pH = 7.4 with NaOH. Drug concentrations were optimized based on previous studies testing dose–response curves to achieve selective blocking effects without off-target activity. Large diameter neurons that display smooth rise in calcium baseline in response to the application of 100 nM kM-RIIIJ were selected for further transcriptomic and electrophysiological characterization.

### Single cell transcriptomics

Single-cell RNA sequencing was performed to analyze the expression profiles of ion channel genes in individual neurons. Individual cells were picked at the end of constellation pharmacology experiments, using fire polished glass capillaries (using Sutter P-90 puller) with a diameter of ~ 3–10 microns. The cells were held at the tip and immediately placed in a microfuge PCR tube containing lysis buffer from Takara Bio Smart-Seq Single Cell kit (Catalog #R400751). The transcriptomic libraries were constructed as per manufacturer protocols and submitted to University of Utah Sequencing core. Sequencing was conducted on Novaseq platform at a depth of 20 million reads per cell, and data were analyzed using bioinformatics pipelines and in–house scripts to identify differentially expressed genes. The single cell transcriptomic data for proprioceptive DRG neurons presented in this study are uploaded to NCBI Gene Expression Omnibus (GEO) with accession number GSE279047.

In order to confirm the transcriptomic identity of individual cells, we created a combined single cell data set by merging our single cell samples with a larger 10X genomics single cell data set (Sharma et al.^24^) using the Seurat package for single cell analysis (https://satijalab.org/seurat/)^25^. Both data sets were corrected for the percentage of mitochondrial reads and for the fraction of reads related to damage repair using the PercentageFeatureSet function and SCTransform function. The 10X single cell data was downsampled to 40 representative cells for each of the cell types. Integration anchors were identified using the FindIntegrationAnchors with dims = 1:30 and the two data sets combined using the IntegrateData function with dims = 1:30. Umap analysis was performed on this data set using the scaled data to confirm co-clustering of our single cell proprioceptors with the larger reference set. Normalized gene expression values reported in Supplementary table 1 are from this data set.

### Whole-cell electrophysiology

Whole-cell patch-clamp recordings were conducted at the end of constellation pharmacology experiments. To measure the membrane potential of neurons, whole-cell current clamp experiments were performed. Cells were bathed in DRG observation solution. Patch pipettes (5–10 MOhm) were filled with an standard internal solution comprising (in mM): 140 K-aspartate, 13.5 NaCl, 1.8 MgCl_2_, 0.09 EGTA, 9 HEPES, 14 creatine phosphate, 4 ATP-Mg, and 0.3 GTP-Tris, pH = 7.2 with KOH. Recordings were made using an Axopatch 200B amplifier, Digidata 1400A, and data were analyzed with Clampfit to determine changes in neuronal firing. To record K-currents, external solution contained 1 μM TTX in DRG observation solution and standard internal solution was used. Patch pipettes ranging from 3.5 MOhm to 5 MOhm were used. To record persistent sodium currents, internal solution contained (in mM): 130 CsCl, 2 MgCl_2_, 10 EGTA, 10 HEPES, 14 creatine phosphate, 4 ATP-Mg, and 0.3 GTP-tris buffered to pH = 7.4 using CsOH. External solution contained (in mM): 155 NaCl, 10 TEA-Cl, 1 4-AP, 2 BaCl_2_, 0.3 CdCl_2_, 10 HEPES, buffered to pH = 7.4 with NaOH. To test the effects of kM-RIIIJ on persistent sodium currents (for Fig. 5), 4-AP was eliminated from external solution. The recordings were performed on standard I-Clamp (not I-clamp fast). Whole-cell GΩ seal was obtained in V-Clamp mode and series resistance was compensated by ~ 70%, which was carried to I-Clamp mode (for bridge balance).

### Voluntary running wheel assays

Six- to eight-week-old mice (23.8 ± 1.2 g) were administered daily intraperitoneal injections of saline solution for four consecutive days. Thirty minutes after each injection, the mice were placed in a chamber equipped with a running wheel connected to an electromagnetic odometer, which recorded wheel rotations digitally. During this period, animals were trained to use the running wheel. On the fifth day, animals were randomly assigned to one of two groups: saline-injected (negative control) or 10 nmol kM-RIIIJ-injected (test group). Thirty minutes after injection, their activity was recorded during a final trial using the same setup. Locomotor activity on day 4 was used as the baseline (100%), and activity on day 5 was normalized to this value. Differences in activity between the control and test groups were analyzed to assess the effect of kM-RIIIJ on locomotion.

### Fish behavioral assay

Groups of four, over 60-day old zebrafish were injected intramuscularly (i.m.) with 12 μL of E3 Buffer – (in mM): 4.96 NaCl, 0.18 KCl, 0.33 CaCl_2_.2H_2_O, 0.4 MgCl_2_•6H_2_O, pH = 7.2 with NaOH—(control group) or the same volume of E3 Buffer containing 5 nmol or 15 nmol of kM-RIIIJ. The injections were performed at the level of the posterior end of the dorsal fin, on the left side of the body. Injections were completed within 1 min and the fish were observed for at least 2 h and the different phenotypes were recorded.

### Statistical analysis

Data are presented as mean ± SEM in graphs. Data are presented as mean ± S.D. in text. Statistical significance was assessed using the Student’s t-test for comparisons between two groups or Wilcoxon matched pairs signed rank test for multiple comparisons. For animal behavior a multiple unpaired t-test was applied. A *p*-value of < 0.05 was considered statistically significant. All analyses were performed using GraphPad Prism software.

## Declaration

## Supplementary Information

Supplementary Information.

## Data Availability

Data is provided within the manuscript and supplementary information files. The single cell transcriptomic data for proprioceptive DRG neurons presented in this study are uploaded to NCBI Gene Expression Omnibus (GEO) with accession number GSE279047.
