# Supplementary material for: Conotoxin kM-RIIIJ reveals interplay between Kv1-channels and persistent sodium currents in proprioceptive DRG neurons
Source: Sci Rep. 2024 Dec 28;14:31001. doi: 10.1038/s41598-024-82165-5 (PMC11681041; doi:10.1038/s41598-024-82165-5)
Supplement: Supplementary file 1 — Supplementary Information. [file 41598_2024_82165_MOESM1_ESM.docx]

**Supplementary data**

**Supplementary Table 1: Transcriptomic data table.** Normalized counts of transcripts from 23 individual proprioceptive neuron.

**Supplementary Table 2: Fish injection – intramuscular**

| Condition | Weight range (g) | Phenotype |
| --- | --- | --- |
| E3 buffer | 0.85 – 1.10 | Quiet at the bottom of the tank for a couple of minutes after injection. Switching between short resting periods and normal swimming. |
| 5 nmol κM-RIIIJ | 0.6 – 0.9 | Hyperactivity (fast swimming, bumping the tank walls) right after injection. Twitching and spasms after 1-5 min. The hyperactivity lasted for 15 - 20 min. Two of the fish were swimming belly-up after 19 and 25 min. Three fish died after 31 - 95 min. |
| 15 nmol κM-RIIIJ | 0.6 – 0.9 | Hyperactivity (fast swimming, bumping the tank walls) right after injection. Twitching and spasms after 1-5 min. Two of the fish were swimming belly-up after 6 and 75 min. Two fish died after 29 and108 min. |


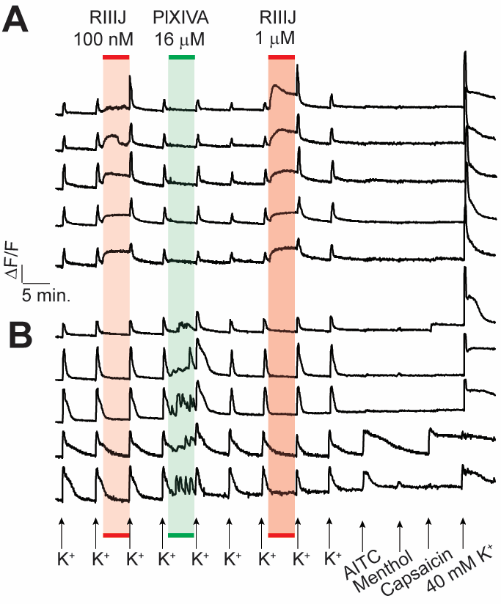


**Figure S1: Expression of K_v_1.2 and K_v_1.6 assessed by conotoxin κM-RIIIJ and κJ-PlXIVA.** Conotoxin κM-RIIIJ and κJ-PlXIVA were tested in the same experiment to monitor the functional expression of K_v_1.2 and K_v_1.6 in cells. **A.** Proprioceptive DRG neurons were sensitive to κM-RIIIJ but not to κJ-PlXIVA, **B.** Representative traces from neurons that were sensitive to κJ-PlXIVA and elicited disturbance in calcium baseline upon κJ-PlXIVA application. However, these neurons did not respond to κM-RIIIJ. These results suggest that K_v_1.2 and K_v_1.6 channels are likely not co-expressed in the same neurons, reducing the possibility of K_v_1.2/1.6 heteromers in proprioceptive DRG neurons.


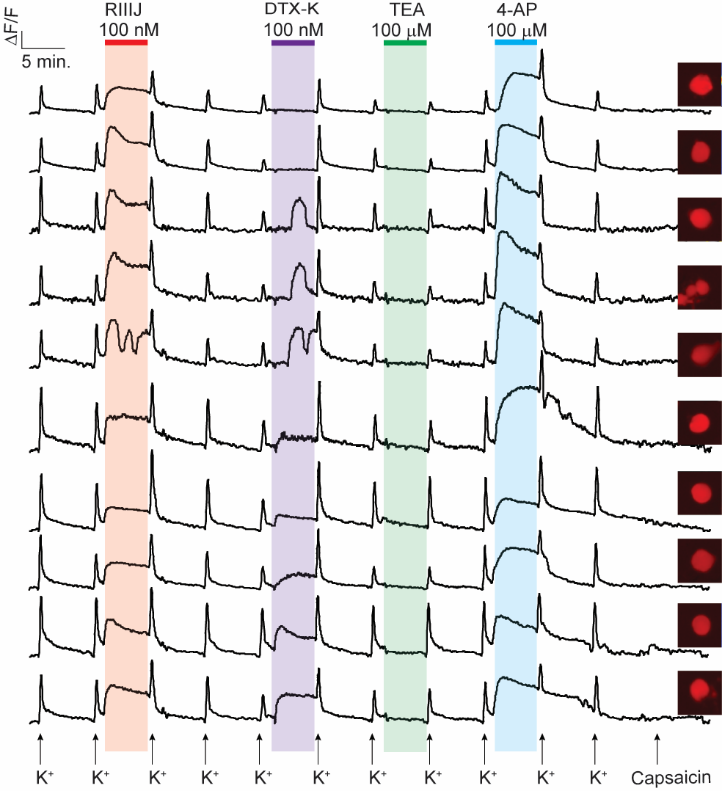


**Figure S2: Expression of K_v_1.2 and K_v_1.1 in proprioceptors assessed by conotoxin κM-RIIIJ and DTX-K respectively.** Representative calcium traces from ten proprioceptive DRG neurons comparing the effects of κM-RIIIJ, dendrotoxin-K, tetraethyl ammonium chloride (TEA) and 4-amino pyridine (4-AP) on neuronal activity.
